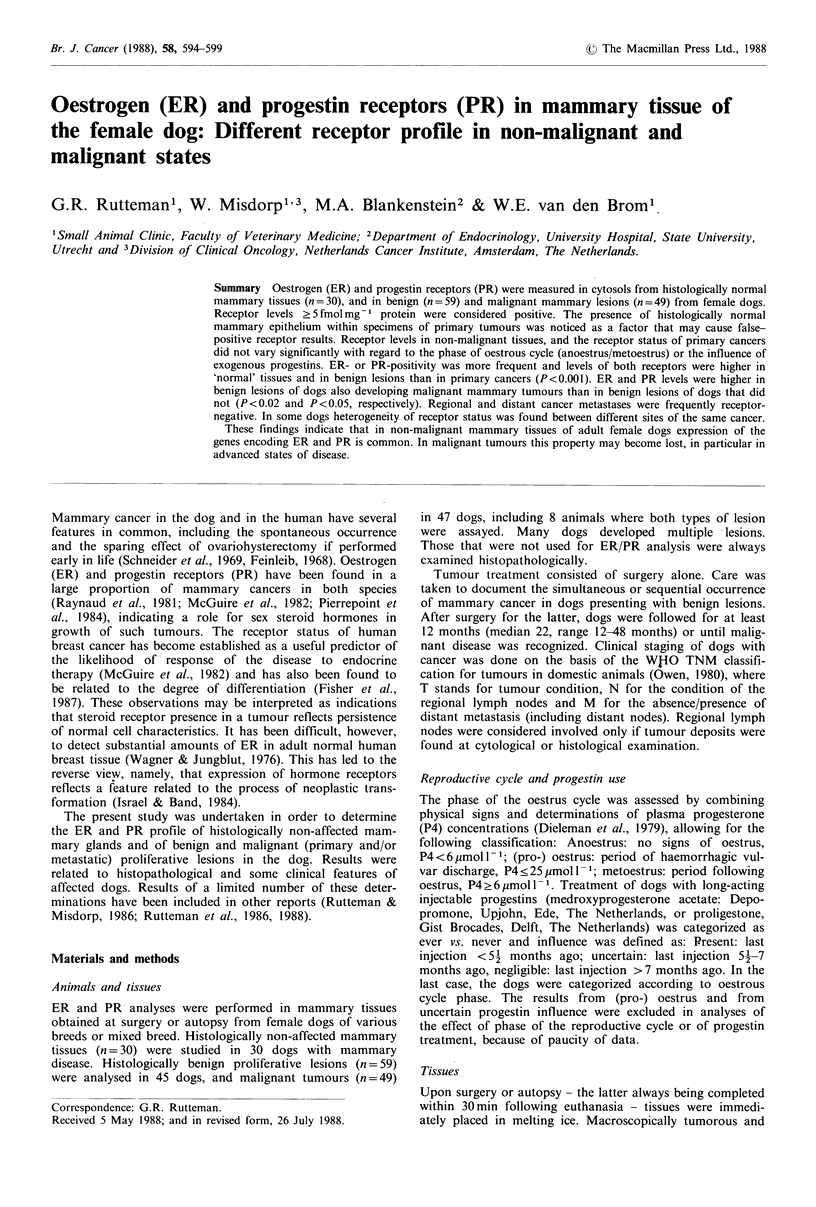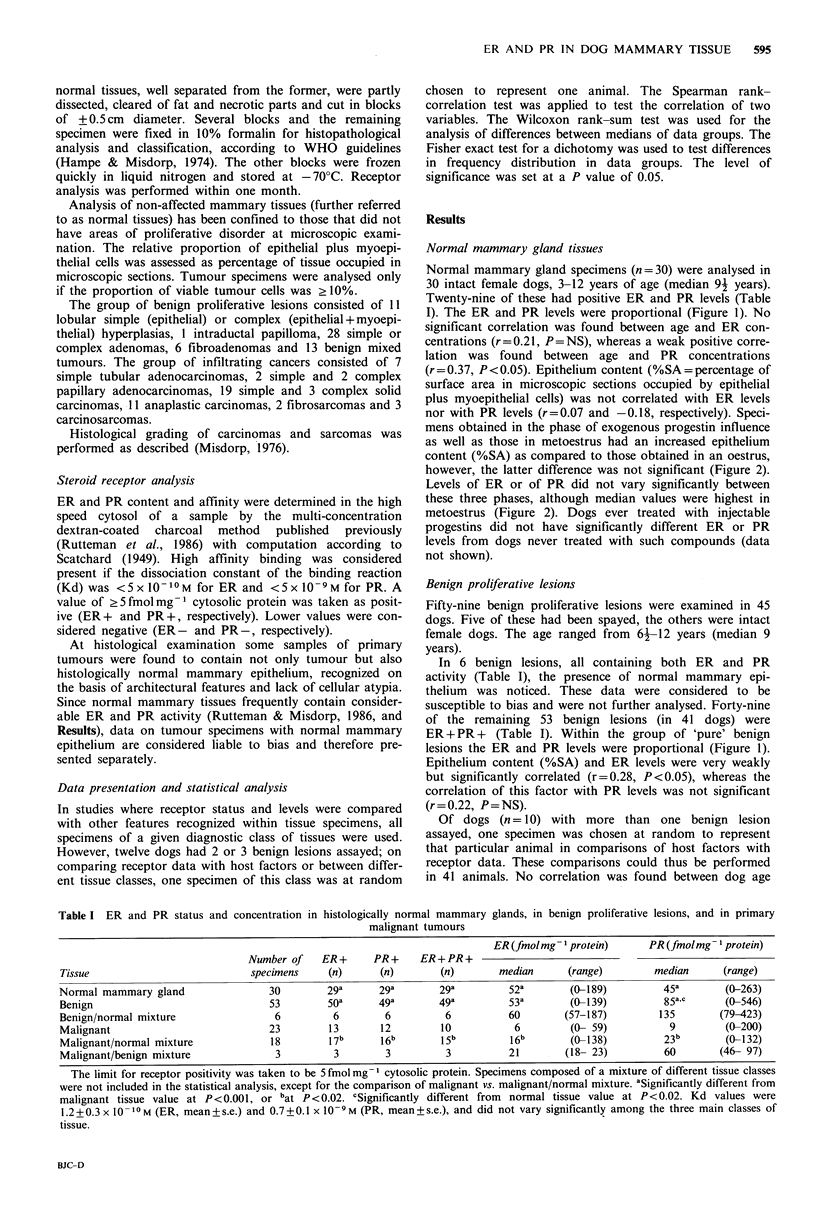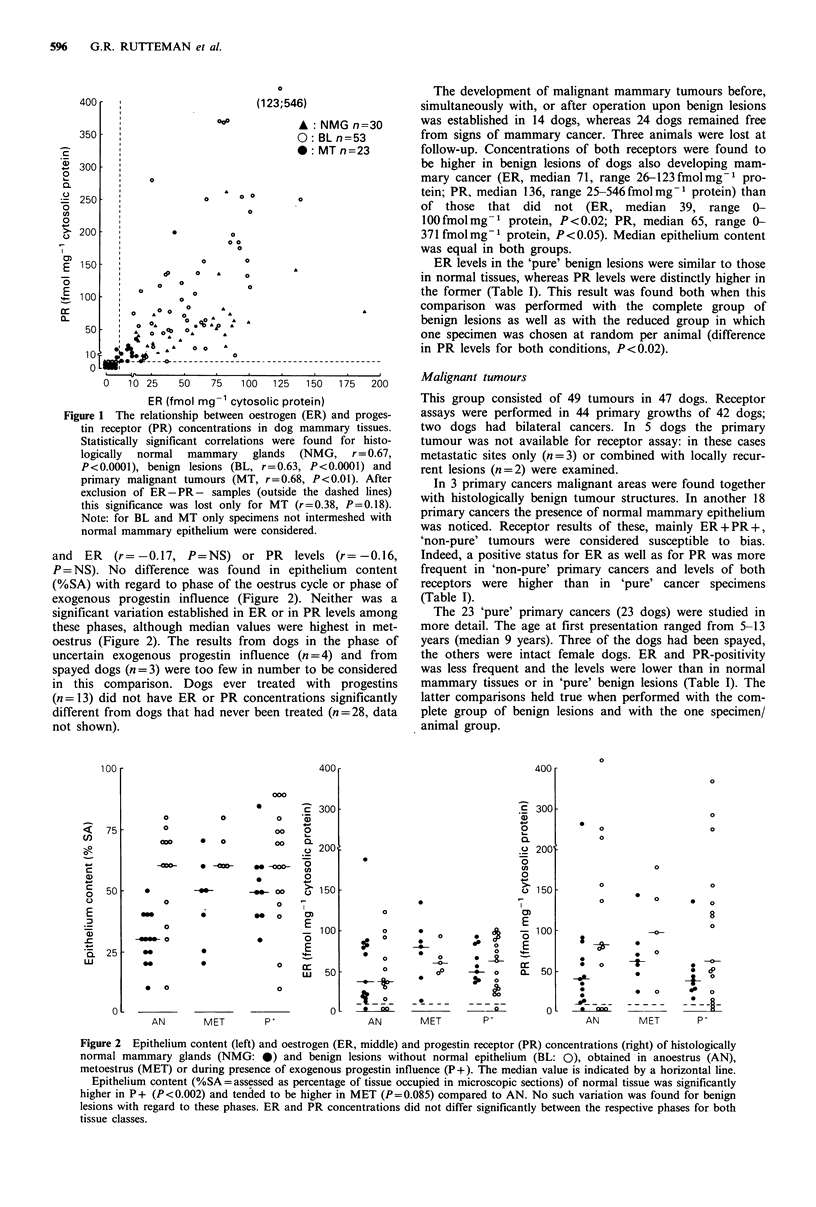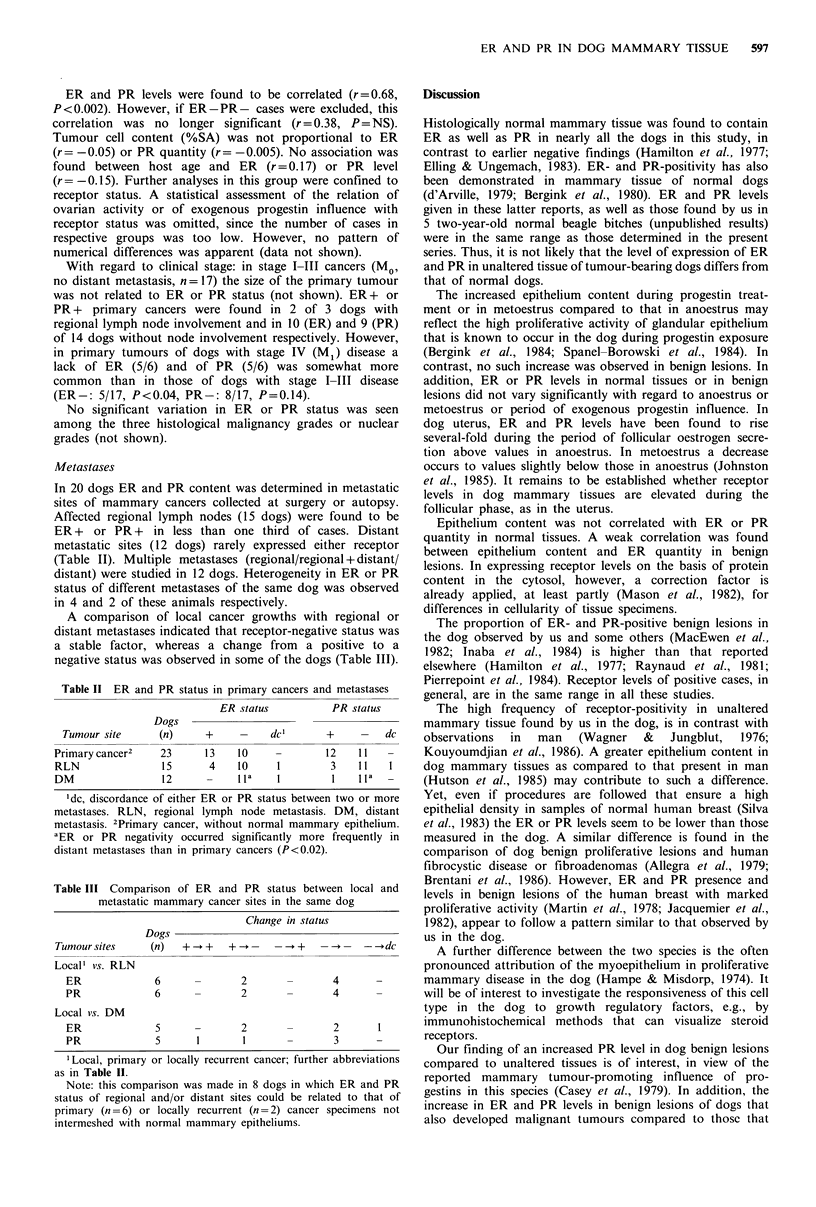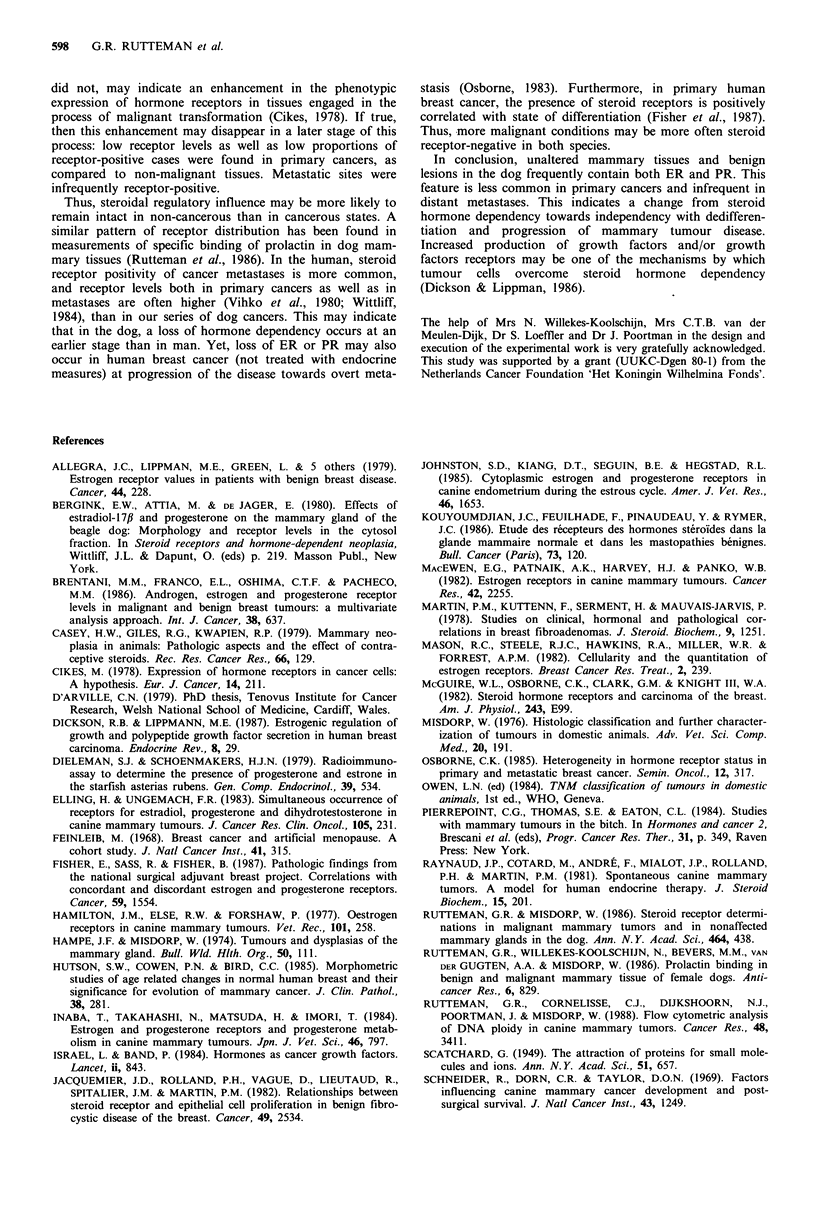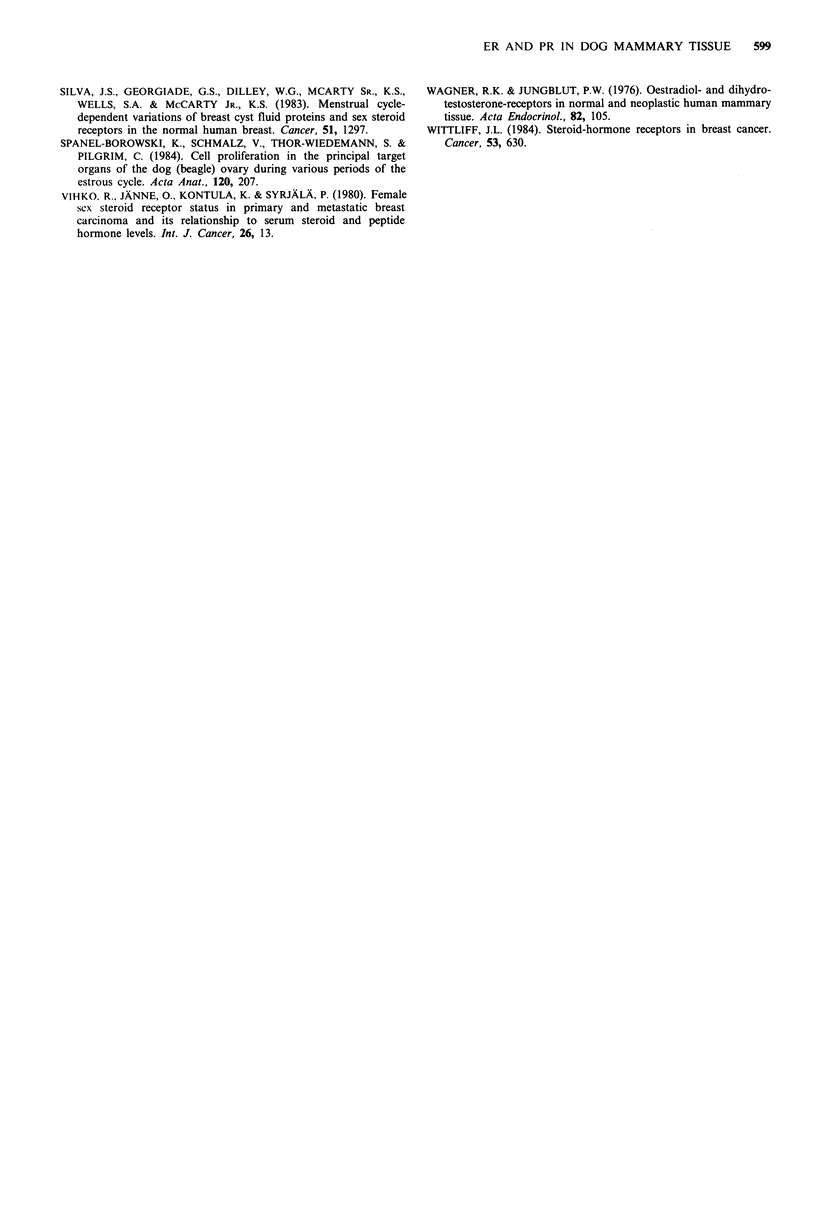# Oestrogen (ER) and progestin receptors (PR) in mammary tissue of the female dog: different receptor profile in non-malignant and malignant states.

**DOI:** 10.1038/bjc.1988.266

**Published:** 1988-11

**Authors:** G. R. Rutteman, W. Misdorp, M. A. Blankenstein, W. E. van den Brom

**Affiliations:** Small Animal Clinic, Faculty of Veterinary Medicine, University Hospital, State University, Utrecht, The Netherlands.

## Abstract

Oestrogen (ER) and progestin receptors (PR) were measured in cytosols from histologically normal mammary tissues (n = 30), and in benign (n = 59) and malignant mammary lesions (n = 49) from female dogs. Receptor levels greater than or equal to 5 fmol mg-1 protein were considered positive. The presence of histologically normal mammary epithelium within specimens of primary tumours was noticed as a factor that may cause false-positive receptor results. Receptor levels in non-malignant tissues, and the receptor status of primary cancers did not vary significantly with regard to the phase of oestrous cycle (anoestrus/metoestrus) or the influence of exogenous progestins. ER- or PR-positivity was more frequent and levels of both receptors were higher in 'normal' tissues and in benign lesions than in primary cancers (P less than 0.001). ER and PR levels were higher in benign lesions of dogs also developing malignant mammary tumours than in benign lesions of dogs that did not (P less than 0.02 and P less than 0.05, respectively). Regional and distant cancer metastases were frequently receptor-negative. In some dogs heterogeneity of receptor status was found between different sites of the same cancer. These findings indicate that in non-malignant mammary tissues of adult female dogs expression of the genes encoding ER and PR is common. In malignant tumours this property may become lost, in particular in advanced states of disease.


Br. J. Cancer (1988), 58, 594–599 © The Macmillan Press Ltd., 1988

### Oestrogen (ER) and progestin receptors (PR) in mammary tissue of the female dog: Different receptor profile in non-malignant and malignant states

G.R. Rutteman[1], W. Misdorp[1,3], M.A. Blankenstein[2] & W.E. van den Brom[1].

[1]*Small Animal Clinic, Faculty of Veterinary Medicine;* [2]*Department of Endocrinology, University Hospital, State University, Utrecht and* [3]*Division of Clinical Oncology, Netherlands Cancer Institute, Amsterdam, The Netherlands.*

**Summary** Oestrogen (ER) and progestin receptors (PR) were measured in cytosols from histologically normal mammary tissues ($n=30$), and in benign ($n=59$) and malignant mammary lesions ($n=49$) from female dogs. Receptor levels $\geq 5\,\mathrm{fmol\,mg^{-1}}$ protein were considered positive. The presence of histologically normal mammary epithelium within specimens of primary tumours was noticed as a factor that may cause false-positive receptor results. Receptor levels in non-malignant tissues, and the receptor status of primary cancers did not vary significantly with regard to the phase of oestrous cycle (anoestrus/metoestrus) or the influence of exogenous progestins. ER- or PR-positivity was more frequent and levels of both receptors were higher in 'normal' tissues and in benign lesions than in primary cancers ($P<0.001$). ER and PR levels were higher in benign lesions of dogs also developing malignant mammary tumours than in benign lesions of dogs that did not ($P<0.02$ and $P<0.05$, respectively). Regional and distant cancer metastases were frequently receptor-negative. In some dogs heterogeneity of receptor status was found between different sites of the same cancer.

These findings indicate that in non-malignant mammary tissues of adult female dogs expression of the genes encoding ER and PR is common. In malignant tumours this property may become lost, in particular in advanced states of disease.

Mammary cancer in the dog and in the human have several features in common, including the spontaneous occurrence and the sparing effect of ovariohysterectomy if performed early in life (Schneider *et al.*, 1969, Feinleib, 1968). Oestrogen (ER) and progestin receptors (PR) have been found in a large proportion of mammary cancers in both species (Raynaud *et al.*, 1981; McGuire *et al.*, 1982; Pierrepoint *et al.*, 1984), indicating a role for sex steroid hormones in growth of such tumours. The receptor status of human breast cancer has become established as a useful predictor of the likelihood of response of the disease to endocrine therapy (McGuire *et al.*, 1982) and has also been found to be related to the degree of differentiation (Fisher *et al.*, 1987). These observations may be interpreted as indications that steroid receptor presence in a tumour reflects persistence of normal cell characteristics. It has been difficult, however, to detect substantial amounts of ER in adult normal human breast tissue (Wagner & Jungblut, 1976). This has led to the reverse view, namely, that expression of hormone receptors reflects a feature related to the process of neoplastic transformation (Israel & Band, 1984).

The present study was undertaken in order to determine the ER and PR profile of histologically non-affected mammary glands and of benign and malignant (primary and/or metastatic) proliferative lesions in the dog. Results were related to histopathological and some clinical features of affected dogs. Results of a limited number of these determinations have been included in other reports (Rutteman & Misdorp, 1986; Rutteman *et al.*, 1986, 1988).

### Materials and methods

### *Animals and tissues*

ER and PR analyses were performed in mammary tissues obtained at surgery or autopsy from female dogs of various breeds or mixed breed. Histologically non-affected mammary tissues ($n=30$) were studied in 30 dogs with mammary disease. Histologically benign proliferative lesions ($n=59$) were analysed in 45 dogs, and malignant tumours ($n=49$) in 47 dogs, including 8 animals where both types of lesion were assayed. Many dogs developed multiple lesions. Those that were not used for ER/PR analysis were always examined histopathologically.

Correspondence: G.R. Rutteman.
Received 5 May 1988; and in revised form, 26 July 1988.

Tumour treatment consisted of surgery alone. Care was taken to document the simultaneous or sequential occurrence of mammary cancer in dogs presenting with benign lesions. After surgery for the latter, dogs were followed for at least 12 months (median 22, range 12–48 months) or until malignant disease was recognized. Clinical staging of dogs with cancer was done on the basis of the WHO TNM classification for tumours in domestic animals (Owen, 1980), where T stands for tumour condition, N for the condition of the regional lymph nodes and M for the absence/presence of distant metastasis (including distant nodes). Regional lymph nodes were considered involved only if tumour deposits were found at cytological or histological examination.

### *Reproductive cycle and progestin use*

The phase of the oestrus cycle was assessed by combining physical signs and determinations of plasma progesterone (P4) concentrations (Dieleman *et al.*, 1979), allowing for the following classification: Anoestrus: no signs of oestrus, $P4<6\,\mu\mathrm{mol\,l^{-1}}$; (pro-) oestrus: period of haemorrhagic vulvar discharge, $P4\leq 25\,\mu\mathrm{mol\,l^{-1}}$; metoestrus: period following oestrus, $P4\geq 6\,\mu\mathrm{mol\,l^{-1}}$. Treatment of dogs with long-acting injectable progestins (medroxyprogesterone acetate: Depo-promone, Upjohn, Ede, The Netherlands, or proligestone, Gist Brocades, Delft, The Netherlands) was categorized as ever *vs.* never and influence was defined as: Present: last injection $<5\frac{1}{2}$ months ago; uncertain: last injection $5\frac{1}{2}$–7 months ago, negligible: last injection $>7$ months ago. In the last case, the dogs were categorized according to oestrous cycle phase. The results from (pro-) oestrus and from uncertain progestin influence were excluded in analyses of the effect of phase of the reproductive cycle or of progestin treatment, because of paucity of data.

### *Tissues*

Upon surgery or autopsy – the latter always being completed within 30 min following euthanasia – tissues were immediately placed in melting ice. Macroscopically tumorous and normal tissues, well separated from the former, were partly dissected, cleared of fat and necrotic parts and cut in blocks of ±0.5 cm diameter. Several blocks and the remaining specimen were fixed in 10% formalin for histopathological analysis and classification, according to WHO guidelines (Hampe & Misdorp, 1974). The other blocks were frozen quickly in liquid nitrogen and stored at −70°C. Receptor analysis was performed within one month.

Analysis of non-affected mammary tissues (further referred to as normal tissues) has been confined to those that did not have areas of proliferative disorder at microscopic examination. The relative proportion of epithelial plus myoepithelial cells was assessed as percentage of tissue occupied in microscopic sections. Tumour specimens were analysed only if the proportion of viable tumour cells was ≥10%.

The group of benign proliferative lesions consisted of 11 lobular simple (epithelial) or complex (epithelial + myoepithelial) hyperplasias, 1 intraductal papilloma, 28 simple or complex adenomas, 6 fibroadenomas and 13 benign mixed tumours. The group of infiltrating cancers consisted of 7 simple tubular adenocarcinomas, 2 simple and 2 complex papillary adenocarcinomas, 19 simple and 3 complex solid carcinomas, 11 anaplastic carcinomas, 2 fibrosarcomas and 3 carcinosarcomas.

Histological grading of carcinomas and sarcomas was performed as described (Misdorp, 1976).

### *Steroid receptor analysis*

ER and PR content and affinity were determined in the high speed cytosol of a sample by the multi-concentration dextran-coated charcoal method published previously (Rutteman *et al.*, 1986) with computation according to Scatchard (1949). High affinity binding was considered present if the dissociation constant of the binding reaction (Kd) was $<5 \times 10^{-10}$ M for ER and $<5 \times 10^{-9}$ M for PR. A value of $\geq 5$ fmol mg$^{-1}$ cytosolic protein was taken as positive (ER+ and PR+, respectively). Lower values were considered negative (ER− and PR−, respectively).

At histological examination some samples of primary tumours were found to contain not only tumour but also histologically normal mammary epithelium, recognized on the basis of architectural features and lack of cellular atypia. Since normal mammary tissues frequently contain considerable ER and PR activity (Rutteman & Misdorp, 1986, and **Results**), data on tumour specimens with normal mammary epithelium are considered liable to bias and therefore presented separately.

### *Data presentation and statistical analysis*

In studies where receptor status and levels were compared with other features recognized within tissue specimens, all specimens of a given diagnostic class of tissues were used. However, twelve dogs had 2 or 3 benign lesions assayed; on comparing receptor data with host factors or between different tissue classes, one specimen of this class was at random chosen to represent one animal. The Spearman rank-correlation test was applied to test the correlation of two variables. The Wilcoxon rank-sum test was used for the analysis of differences between medians of data groups. The Fisher exact test for a dichotomy was used to test differences in frequency distribution in data groups. The level of significance was set at a $P$ value of 0.05.

### Results

### *Normal mammary gland tissues*

Normal mammary gland specimens ($n=30$) were analysed in 30 intact female dogs, 3–12 years of age (median 9½ years). Twenty-nine of these had positive ER and PR levels (Table I). The ER and PR levels were proportional (Figure 1). No significant correlation was found between age and ER concentrations ($r=0.21$, $P=$NS), whereas a weak positive correlation was found between age and PR concentrations ($r=0.37$, $P<0.05$). Epithelium content (%SA = percentage of surface area in microscopic sections occupied by epithelial plus myoepithelial cells) was not correlated with ER levels nor with PR levels ($r=0.07$ and $-0.18$, respectively). Specimens obtained in the phase of exogenous progestin influence as well as those in metoestrus had an increased epithelium content (%SA) as compared to those obtained in an oestrus, however, the latter difference was not significant (Figure 2). Levels of ER or of PR did not vary significantly between these three phases, although median values were highest in metoestrus (Figure 2). Dogs ever treated with injectable progestins did not have significantly different ER or PR levels from dogs never treated with such compounds (data not shown).

### *Benign proliferative lesions*

Fifty-nine benign proliferative lesions were examined in 45 dogs. Five of these had been spayed, the others were intact female dogs. The age ranged from 6½–12 years (median 9 years).

In 6 benign lesions, all containing both ER and PR activity (Table I), the presence of normal mammary epithelium was noticed. These data were considered to be susceptible to bias and were not further analysed. Forty-nine of the remaining 53 benign lesions (in 41 dogs) were ER+PR+ (Table I). Within the group of 'pure' benign lesions the ER and PR levels were proportional (Figure 1). Epithelium content (%SA) and ER levels were very weakly but significantly correlated ($r=0.28$, $P<0.05$), whereas the correlation of this factor with PR levels was not significant ($r=0.22$, $P=$NS).

Of dogs ($n=10$) with more than one benign lesion assayed, one specimen was chosen at random to represent that particular animal in comparisons of host factors with receptor data. These comparisons could thus be performed in 41 animals. No correlation was found between dog age

**Table I** ER and PR status and concentration in histologically normal mammary glands, in benign proliferative lesions, and in primary malignant tumours

| Tissue | Number of specimens | ER+ (n) | PR+ (n) | ER+PR+ (n) | ER (fmol mg$^{-1}$ protein) median | (range) | PR (fmol mg$^{-1}$ protein) median | (range) |
|---|---|---|---|---|---|---|---|---|
| Normal mammary gland | 30 | 29[a] | 29[a] | 29[a] | 52[a] | (0–189) | 45[a] | (0–263) |
| Benign | 53 | 50[a] | 49[a] | 49[a] | 53[a] | (0–139) | 85[a,c] | (0–546) |
| Benign/normal mixture | 6 | 6 | 6 | 6 | 60 | (57–187) | 135 | (79–423) |
| Malignant | 23 | 13 | 12 | 10 | 6 | (0– 59) | 9 | (0–200) |
| Malignant/normal mixture | 18 | 17[b] | 16[b] | 15[b] | 16[b] | (0–138) | 23[b] | (0–132) |
| Malignant/benign mixture | 3 | 3 | 3 | 3 | 21 | (18– 23) | 60 | (46– 97) |

The limit for receptor positivity was taken to be 5 fmol mg$^{-1}$ cytosolic protein. Specimens composed of a mixture of different tissue classes were not included in the statistical analysis, except for the comparison of malignant *vs.* malignant/normal mixture. [a]Significantly different from malignant tissue value at $P<0.001$, or [b]at $P<0.02$. [c]Significantly different from normal tissue value at $P<0.02$. Kd values were $1.2 \pm 0.3 \times 10^{-10}$ M (ER, mean ± s.e.) and $0.7 \pm 0.1 \times 10^{-9}$ M (PR, mean ± s.e.), and did not vary significantly among the three main classes of tissue.

**Figure 1** The relationship between oestrogen (ER) and progestin receptor (PR) concentrations in dog mammary tissues. Statistically significant correlations were found for histologically normal mammary glands (NMG, $r=0.67$, $P<0.0001$), benign lesions (BL, $r=0.63$, $P<0.0001$) and primary malignant tumours (MT, $r=0.68$, $P<0.01$). After exclusion of ER−PR− samples (outside the dashed lines) this significance was lost only for MT ($r=0.38$, $P=0.18$). Note: for BL and MT only specimens not intermeshed with normal mammary epithelium were considered.

and ER ($r=-0.17$, $P=$NS) or PR levels ($r=-0.16$, $P=$NS). No difference was found in epithelium content (%SA) with regard to phase of the oestrus cycle or phase of exogenous progestin influence (Figure 2). Neither was a significant variation established in ER or in PR levels among these phases, although median values were highest in metoestrus (Figure 2). The results from dogs in the phase of uncertain exogenous progestin influence ($n=4$) and from spayed dogs ($n=3$) were too few in number to be considered in this comparison. Dogs ever treated with progestins ($n=13$) did not have ER or PR concentrations significantly different from dogs that had never been treated ($n=28$, data not shown).

The development of malignant mammary tumours before, simultaneously with, or after operation upon benign lesions was established in 14 dogs, whereas 24 dogs remained free from signs of mammary cancer. Three animals were lost at follow-up. Concentrations of both receptors were found to be higher in benign lesions of dogs also developing mammary cancer (ER, median 71, range 26–123 fmol mg$^{-1}$ protein; PR, median 136, range 25–546 fmol mg$^{-1}$ protein) than of those that did not (ER, median 39, range 0–100 fmol mg$^{-1}$ protein, $P<0.02$; PR, median 65, range 0–371 fmol mg$^{-1}$ protein, $P<0.05$). Median epithelium content was equal in both groups.

ER levels in the 'pure' benign lesions were similar to those in normal tissues, whereas PR levels were distinctly higher in the former (Table I). This result was found both when this comparison was performed with the complete group of benign lesions as well as with the reduced group in which one specimen was chosen at random per animal (difference in PR levels for both conditions, $P<0.02$).

*Malignant tumours*

This group consisted of 49 tumours in 47 dogs. Receptor assays were performed in 44 primary growths of 42 dogs; two dogs had bilateral cancers. In 5 dogs the primary tumour was not available for receptor assay: in these cases metastatic sites only ($n=3$) or combined with locally recurrent lesions ($n=2$) were examined.

In 3 primary cancers malignant areas were found together with histologically benign tumour structures. In another 18 primary cancers the presence of normal mammary epithelium was noticed. Receptor results of these, mainly ER+PR+, 'non-pure' tumours were considered susceptible to bias. Indeed, a positive status for ER as well as for PR was more frequent in 'non-pure' primary cancers and levels of both receptors were higher than in 'pure' cancer specimens (Table I).

The 23 'pure' primary cancers (23 dogs) were studied in more detail. The age at first presentation ranged from 5–13 years (median 9 years). Three of the dogs had been spayed, the others were intact female dogs. ER and PR-positivity was less frequent and the levels were lower than in normal mammary tissues or in 'pure' benign lesions (Table I). The latter comparisons held true when performed with the complete group of benign lesions and with the one specimen/animal group.

**Figure 2** Epithelium content (left) and oestrogen (ER, middle) and progestin receptor (PR) concentrations (right) of histologically normal mammary glands (NMG: ●) and benign lesions without normal epithelium (BL: ○), obtained in anoestrus (AN), metoestrus (MET) or during presence of exogenous progestin influence (P+). The median value is indicated by a horizontal line. Epithelium content (%SA = assessed as percentage of tissue occupied in microscopic sections) of normal tissue was significantly higher in P+ ($P<0.002$) and tended to be higher in MET ($P=0.085$) compared to AN. No such variation was found for benign lesions with regard to these phases. ER and PR concentrations did not differ significantly between the respective phases for both tissue classes.

ER and PR levels were found to be correlated ($r=0.68$, $P<0.002$). However, if ER−PR− cases were excluded, this correlation was no longer significant ($r=0.38$, $P$=NS). Tumour cell content (%SA) was not proportional to ER ($r=-0.05$) or PR quantity ($r=-0.005$). No association was found between host age and ER ($r=0.17$) or PR level ($r=-0.15$). Further analyses in this group were confined to receptor status. A statistical assessment of the relation of ovarian activity or of exogenous progestin influence with receptor status was omitted, since the number of cases in respective groups was too low. However, no pattern of numerical differences was apparent (data not shown).

With regard to clinical stage: in stage I–III cancers ($M_0$, no distant metastasis, $n=17$) the size of the primary tumour was not related to ER or PR status (not shown). ER+ or PR+ primary cancers were found in 2 of 3 dogs with regional lymph node involvement and in 10 (ER) and 9 (PR) of 14 dogs without node involvement respectively. However, in primary tumours of dogs with stage IV ($M_1$) disease a lack of ER (5/6) and of PR (5/6) was somewhat more common than in those of dogs with stage I–III disease (ER−: 5/17, $P<0.04$, PR−: 8/17, $P=0.14$).

No significant variation in ER or PR status was seen among the three histological malignancy grades or nuclear grades (not shown).

*Metastases*

In 20 dogs ER and PR content was determined in metastatic sites of mammary cancers collected at surgery or autopsy. Affected regional lymph nodes (15 dogs) were found to be ER+ or PR+ in less than one third of cases. Distant metastatic sites (12 dogs) rarely expressed either receptor (Table II). Multiple metastases (regional/regional+distant/distant) were studied in 12 dogs. Heterogeneity in ER or PR status of different metastases of the same dog was observed in 4 and 2 of these animals respectively.

A comparison of local cancer growths with regional or distant metastases indicated that receptor-negative status was a stable factor, whereas a change from a positive to a negative status was observed in some of the dogs (Table III).

**Table II** ER and PR status in primary cancers and metastases

| Tumour site | Dogs (*n*) | *ER status* + | – | dc[1] | *PR status* + | – | dc |
|---|---|---|---|---|---|---|---|
| Primary cancer[2] | 23 | 13 | 10 | – | 12 | 11 | – |
| RLN | 15 | 4 | 10 | 1 | 3 | 11 | 1 |
| DM | 12 | – | 11[a] | 1 | 1 | 11[a] | – |

[1]dc, discordance of either ER or PR status between two or more metastases. RLN, regional lymph node metastasis. DM, distant metastasis. [2]Primary cancer, without normal mammary epithelium. [a]ER or PR negativity occurred significantly more frequently in distant metastases than in primary cancers ($P<0.02$).

**Table III** Comparison of ER and PR status between local and metastatic mammary cancer sites in the same dog

| Tumour sites | Dogs (*n*) | *Change in status* +→+ | +→– | –→+ | –→– | –→dc |
|---|---|---|---|---|---|---|
| Local[1] *vs*. RLN | | | | | | |
| ER | 6 | – | 2 | – | 4 | – |
| PR | 6 | – | 2 | – | 4 | – |
| Local *vs*. DM | | | | | | |
| ER | 5 | – | 2 | – | 2 | 1 |
| PR | 5 | 1 | 1 | – | 3 | – |

[1]Local, primary or locally recurrent cancer; further abbreviations as in **Table II**.

Note: this comparison was made in 8 dogs in which ER and PR status of regional and/or distant sites could be related to that of primary ($n=6$) or locally recurrent ($n=2$) cancer specimens not intermeshed with normal mammary epitheliums.

### Discussion

Histologically normal mammary tissue was found to contain ER as well as PR in nearly all the dogs in this study, in contrast to earlier negative findings (Hamilton *et al.*, 1977; Elling & Ungemach, 1983). ER- and PR-positivity has also been demonstrated in mammary tissue of normal dogs (d'Arville, 1979; Bergink *et al.*, 1980). ER and PR levels given in these latter reports, as well as those found by us in 5 two-year-old normal beagle bitches (unpublished results) were in the same range as those determined in the present series. Thus, it is not likely that the level of expression of ER and PR in unaltered tissue of tumour-bearing dogs differs from that of normal dogs.

The increased epithelium content during progestin treatment or in metoestrus compared to that in anoestrus may reflect the high proliferative activity of glandular epithelium that is known to occur in the dog during progestin exposure (Bergink *et al.*, 1984; Spanel-Borowski *et al.*, 1984). In contrast, no such increase was observed in benign lesions. In addition, ER or PR levels in normal tissues or in benign lesions did not vary significantly with regard to anoestrus or metoestrus or period of exogenous progestin influence. In dog uterus, ER and PR levels have been found to rise several-fold during the period of follicular oestrogen secretion above values in anoestrus. In metoestrus a decrease occurs to values slightly below those in anoestrus (Johnston *et al.*, 1985). It remains to be established whether receptor levels in dog mammary tissues are elevated during the follicular phase, as in the uterus.

Epithelium content was not correlated with ER or PR quantity in normal tissues. A weak correlation was found between epithelium content and ER quantity in benign lesions. In expressing receptor levels on the basis of protein content in the cytosol, however, a correction factor is already applied, at least partly (Mason *et al.*, 1982), for differences in cellularity of tissue specimens.

The proportion of ER- and PR-positive benign lesions in the dog observed by us and some others (MacEwen *et al.*, 1982; Inaba *et al.*, 1984) is higher than that reported elsewhere (Hamilton *et al.*, 1977; Raynaud *et al.*, 1981; Pierrepoint *et al.*, 1984). Receptor levels of positive cases, in general, are in the same range in all these studies.

The high frequency of receptor-positivity in unaltered mammary tissue found by us in the dog, is in contrast with observations in man (Wagner & Jungblut, 1976; Kouyoumdjian *et al.*, 1986). A greater epithelium content in dog mammary tissues as compared to that present in man (Hutson *et al.*, 1985) may contribute to such a difference. Yet, even if procedures are followed that ensure a high epithelial density in samples of normal human breast (Silva *et al.*, 1983) the ER or PR levels seem to be lower than those measured in the dog. A similar difference is found in the comparison of dog benign proliferative lesions and human fibrocystic disease or fibroadenomas (Allegra *et al.*, 1979; Brentani *et al.*, 1986). However, ER and PR presence and levels in benign lesions of the human breast with marked proliferative activity (Martin *et al.*, 1978; Jacquemier *et al.*, 1982), appear to follow a pattern similar to that observed by us in the dog.

A further difference between the two species is the often pronounced attribution of the myoepithelium in proliferative mammary disease in the dog (Hampe & Misdorp, 1974). It will be of interest to investigate the responsiveness of this cell type in the dog to growth regulatory factors, e.g., by immunohistochemical methods that can visualize steroid receptors.

Our finding of an increased PR level in dog benign lesions compared to unaltered tissues is of interest, in view of the reported mammary tumour-promoting influence of progestins in this species (Casey *et al.*, 1979). In addition, the increase in ER and PR levels in benign lesions of dogs that also developed malignant tumours compared to those that did not, may indicate an enhancement in the phenotypic expression of hormone receptors in tissues engaged in the process of malignant transformation (Cikes, 1978). If true, then this enhancement may disappear in a later stage of this process: low receptor levels as well as low proportions of receptor-positive cases were found in primary cancers, as compared to non-malignant tissues. Metastatic sites were infrequently receptor-positive.

Thus, steroidal regulatory influence may be more likely to remain intact in non-cancerous than in cancerous states. A similar pattern of receptor distribution has been found in measurements of specific binding of prolactin in dog mammary tissues (Rutteman *et al.*, 1986). In the human, steroid receptor positivity of cancer metastases is more common, and receptor levels both in primary cancers as well as in metastases are often higher (Vihko *et al.*, 1980; Wittliff, 1984), than in our series of dog cancers. This may indicate that in the dog, a loss of hormone dependency occurs at an earlier stage than in man. Yet, loss of ER or PR may also occur in human breast cancer (not treated with endocrine measures) at progression of the disease towards overt metastasis (Osborne, 1983). Furthermore, in primary human breast cancer, the presence of steroid receptors is positively correlated with state of differentiation (Fisher *et al.*, 1987). Thus, more malignant conditions may be more often steroid receptor-negative in both species.

In conclusion, unaltered mammary tissues and benign lesions in the dog frequently contain both ER and PR. This feature is less common in primary cancers and infrequent in distant metastases. This indicates a change from steroid hormone dependency towards independency with dedifferentiation and progression of mammary tumour disease. Increased production of growth factors and/or growth factors receptors may be one of the mechanisms by which tumour cells overcome steroid hormone dependency (Dickson & Lippman, 1986).

The help of Mrs N. Willekes-Koolschijn, Mrs C.T.B. van der Meulen-Dijk, Dr S. Loeffler and Dr J. Poortman in the design and execution of the experimental work is very gratefully acknowledged. This study was supported by a grant (UUKC-Dgen 80-1) from the Netherlands Cancer Foundation 'Het Koningin Wilhelmina Fonds'.

### References

ALLEGRA, J.C., LIPPMAN, M.E., GREEN, L. & 5 others (1979). Estrogen receptor values in patients with benign breast disease. *Cancer*, **44**, 228.

BERGINK, E.W., ATTIA, M. & DE JAGER, E. (1980). Effects of estradiol-17$\beta$ and progesterone on the mammary gland of the beagle dog: Morphology and receptor levels in the cytosol fraction. In *Steroid receptors and hormone-dependent neoplasia*, Wittliff, J.L. & Dapunt, O. (eds) p. 219. Masson Publ., New York.

BRENTANI, M.M., FRANCO, E.L., OSHIMA, C.T.F. & PACHECO, M.M. (1986). Androgen, estrogen and progesterone receptor levels in malignant and benign breast tumours: a multivariate analysis approach. *Int. J. Cancer*, **38**, 637.

CASEY, H.W., GILES, R.G., KWAPIEN, R.P. (1979). Mammary neoplasia in animals: Pathologic aspects and the effect of contraceptive steroids. *Rec. Res. Cancer Res.*, **66**, 129.

CIKES, M. (1978). Expression of hormone receptors in cancer cells: A hypothesis. *Eur. J. Cancer*, **14**, 211.

D'ARVILLE, C.N. (1979). PhD thesis, Tenovus Institute for Cancer Research, Welsh National School of Medicine, Cardiff, Wales.

DICKSON, R.B. & LIPPMANN, M.E. (1987). Estrogenic regulation of growth and polypeptide growth factor secretion in human breast carcinoma. *Endocrine Rev.*, **8**, 29.

DIELEMAN, S.J. & SCHOENMAKERS, H.J.N. (1979). Radioimmunoassay to determine the presence of progesterone and estrone in the starfish asterias rubens. *Gen. Comp. Endocrinol.*, **39**, 534.

ELLING, H. & UNGEMACH, F.R. (1983). Simultaneous occurrence of receptors for estradiol, progesterone and dihydrotestosterone in canine mammary tumours. *J. Cancer Res. Clin. Oncol.*, **105**, 231.

FEINLEIB, M. (1968). Breast cancer and artificial menopause. A cohort study. *J. Natl Cancer Inst.*, **41**, 315.

FISHER, E., SASS, R. & FISHER, B. (1987). Pathologic findings from the national surgical adjuvant breast project. Correlations with concordant and discordant estrogen and progesterone receptors. *Cancer*, **59**, 1554.

HAMILTON, J.M., ELSE, R.W. & FORSHAW, P. (1977). Oestrogen receptors in canine mammary tumours. *Vet. Rec.*, **101**, 258.

HAMPE, J.F. & MISDORP, W. (1974). Tumours and dysplasias of the mammary gland. *Bull. Wld. Hlth. Org.*, **50**, 111.

HUTSON, S.W., COWEN, P.N. & BIRD, C.C. (1985). Morphometric studies of age related changes in normal human breast and their significance for evolution of mammary cancer. *J. Clin. Pathol.*, **38**, 281.

INABA, T., TAKAHASHI, N., MATSUDA, H. & IMORI, T. (1984). Estrogen and progesterone receptors and progesterone metabolism in canine mammary tumours. *Jpn. J. Vet. Sci.*, **46**, 797.

ISRAEL, L. & BAND, P. (1984). Hormones as cancer growth factors. *Lancet*, **ii**, 843.

JACQUEMIER, J.D., ROLLAND, P.H., VAGUE, D., LIEUTAUD, R., SPITALIER, J.M. & MARTIN, P.M. (1982). Relationships between steroid receptor and epithelial cell proliferation in benign fibrocystic disease of the breast. *Cancer*, **49**, 2534.

JOHNSTON, S.D., KIANG, D.T., SEGUIN, B.E. & HEGSTAD, R.L. (1985). Cytoplasmic estrogen and progesterone receptors in canine endometrium during the estrous cycle. *Amer. J. Vet. Res.*, **46**, 1653.

KOUYOUMDJIAN, J.C., FEUILHADE, F., PINAUDEAU, Y. & RYMER, J.C. (1986). Etude des récepteurs des hormones stéroïdes dans la glande mammaire normale et dans les mastopathies bénignes. *Bull. Cancer (Paris)*, **73**, 120.

MACEWEN, E.G., PATNAIK, A.K., HARVEY, H.J. & PANKO, W.B. (1982). Estrogen receptors in canine mammary tumours. *Cancer Res.*, **42**, 2255.

MARTIN, P.M., KUTTENN, F., SERMENT, H. & MAUVAIS-JARVIS, P. (1978). Studies on clinical, hormonal and pathological correlations in breast fibroadenomas. *J. Steroid. Biochem.*, **9**, 1251.

MASON, R.C., STEELE, R.J.C., HAWKINS, R.A., MILLER, W.R. & FORREST, A.P.M. (1982). Cellularity and the quantitation of estrogen receptors. *Breast Cancer Res. Treat.*, **2**, 239.

MCGUIRE, W.L., OSBORNE, C.K., CLARK, G.M. & KNIGHT III, W.A. (1982). Steroid hormone receptors and carcinoma of the breast. *Am. J. Physiol.*, **243**, E99.

MISDORP, W. (1976). Histologic classification and further characterization of tumours in domestic animals. *Adv. Vet. Sci. Comp. Med.*, **20**, 191.

OSBORNE, C.K. (1985). Heterogeneity in hormone receptor status in primary and metastatic breast cancer. *Semin. Oncol.*, **12**, 317.

OWEN, L.N. (ed) (1984). *TNM classification of tumours in domestic animals*, 1st ed., WHO, Geneva.

PIERREPOINT, C.G., THOMAS, S.E. & EATON, C.L. (1984). Studies with mammary tumours in the bitch. In *Hormones and cancer 2*, Brescani *et al.* (eds), *Progr. Cancer Res. Ther.*, **31**, p. 349, Raven Press: New York.

RAYNAUD, J.P., COTARD, M., ANDRÉ, F., MIALOT, J.P., ROLLAND, P.H. & MARTIN, P.M. (1981). Spontaneous canine mammary tumors. A model for human endocrine therapy. *J. Steroid Biochem.*, **15**, 201.

RUTTEMAN, G.R. & MISDORP, W. (1986). Steroid receptor determinations in malignant mammary tumors and in nonaffected mammary glands in the dog. *Ann. N.Y. Acad. Sci.*, **464**, 438.

RUTTEMAN, G.R., WILLEKES-KOOLSCHIJN, N., BEVERS, M.M., VAN DER GUGTEN, A.A. & MISDORP, W. (1986). Prolactin binding in benign and malignant mammary tissue of female dogs. *Anticancer Res.*, **6**, 829.

RUTTEMAN, G.R., CORNELISSE, C.J., DIJKSHOORN, N.J., POORTMAN, J. & MISDORP, W. (1988). Flow cytometric analysis of DNA ploidy in canine mammary tumors. *Cancer Res.*, **48**, 3411.

SCATCHARD, G. (1949). The attraction of proteins for small molecules and ions. *Ann. N.Y. Acad. Sci.*, **51**, 657.

SCHNEIDER, R., DORN, C.R. & TAYLOR, D.O.N. (1969). Factors influencing canine mammary cancer development and postsurgical survival. *J. Natl Cancer Inst.*, **43**, 1249.

SILVA, J.S., GEORGIADE, G.S., DILLEY, W.G., MCARTY SR., K.S., WELLS, S.A. & McCARTY JR., K.S. (1983). Menstrual cycle-dependent variations of breast cyst fluid proteins and sex steroid receptors in the normal human breast. *Cancer*, **51**, 1297.

SPANEL-BOROWSKI, K., SCHMALZ, V., THOR-WIEDEMANN, S. & PILGRIM, C. (1984). Cell proliferation in the principal target organs of the dog (beagle) ovary during various periods of the estrous cycle. *Acta Anat.*, **120**, 207.

VIHKO, R., JÄNNE, O., KONTULA, K. & SYRJÄLÄ, P. (1980). Female sex steroid receptor status in primary and metastatic breast carcinoma and its relationship to serum steroid and peptide hormone levels. *Int. J. Cancer*, **26**, 13.

WAGNER, R.K. & JUNGBLUT, P.W. (1976). Oestradiol- and dihydrotestosterone-receptors in normal and neoplastic human mammary tissue. *Acta Endocrinol.*, **82**, 105.

WITTLIFF, J.L. (1984). Steroid-hormone receptors in breast cancer. *Cancer*, **53**, 630.